# Preliminary Evaluation of the Virtual Reality–Based Gait Sensory Interaction Test (GaitSIT) for Quantifying Sensory Reweighting During Walking Balance

**DOI:** 10.3390/mps9040108

**Published:** 2026-07-09

**Authors:** Priyo Ranjan Kundu Prosun, Shafique Chaudhry, Masudul H. Imtiaz, Poorna Raavi, David DiSalvo, Kwadwo O. Appiah-Kubi

**Affiliations:** 1 Department of Computer Science, Clarkson University, Potsdam, NY 13699, USA; prosunp@clarkson.edu; 2 Reh School of Business, Clarkson University, Potsdam, NY 13699, USA; schaudhr@clarkson.edu; 3 Department of Electrical and Computer Engineering, Clarkson University, Potsdam, NY 13699, USA; mimtiaz@clarkson.edu (M.H.I.); raavip@clarkson.edu (P.R.); 4 Department of Physical Therapy, Clarkson University, Potsdam, NY 13699, USA; ddisalvo@clarkson.edu

**Keywords:** virtualreality, gait–balance, Gait Sensory Interaction Test, sensory reweighting, sensory integration, head-mounted display, Sensory Organization Test, modified Clinical Test of Sensory Interaction on Balance

## Abstract

**Background:** Walking is a dynamic activity that relies on inputs from the multisensory system, i.e., somatosensory, vision, and vestibular. These inputs are processed and integrated in the central nervous system to produce motor impulses for efficient walking balance. The Sensory Organization Test (SOT) is established as the gold standard for assessing sensory contributions to standing balance. However, no comparable assessments have been developed for the clinical evaluation of balance during gait. This study evaluated the Gait Sensory Interaction Test (GaitSIT), a novel virtual reality (VR)-based assessment for characterizing sensory-condition-specific changes in walking balance. **Methods:** The GaitSIT comprises a VR environment with a physical compliant foam walking surface that evaluates gait–balance by systematically manipulating and evaluating the sensory systems. Twenty-nine healthy young adults (mean age 24.9 ± 6.4 years) were instructed to complete 6 m walking trials under six standardized conditions (C): eyes open, eyes closed/dark scene, and rotating visual scenes on a firm surface, then repeated on a foam surface. Wearing an Oculus VR headset, participants were instructed to walk in a straight line at their preferred speed, as naturally as possible, in two test sessions on the same day, followed by a third test session 24 h later. Headset-derived sway measures, including position, velocity, and acceleration data, were recorded, and the continuous trajectory deviation angle (i.e., directional control) and sensory ratios were calculated. Linear mixed-effects models included trial-level walking speed as a covariate. Additionally, participants completed the modified Clinical Test of Sensory Interaction on Balance (mCTSIB) as a clinical standing-balance reference measure; its concurrent-validity findings will be reported separately. **Results:** Significant condition effects were observed for position, velocity, acceleration, and CTDA after adjustment for trial-level walking speed (all p<0.001), indicating that the six sensory conditions elicited distinct gait–balance responses. Significant differences relative to the baseline condition (C1) were observed across conditions C2–C6 for position, C3–C6 for velocity, and C2 and C5 for acceleration. Session effects were not significant for any primary kinematic outcome after speed adjustment. A significant condition × session interaction was observed for position (p<0.001), whereas velocity, acceleration, and CTDA demonstrated no significant interactions. Walking speed was significantly associated with position, acceleration, and CTDA, but not velocity. Sensory-ratio analyses revealed larger visual and vestibular ratios relative to somatosensory ratios, with the visual and vestibular ratios generally decreasing across sessions. **Conclusions:** GaitSIT successfully manipulated sensory conditions during overground walking and produced significant changes in gait-related sway, directional control, and sensory-ratio measures. These findings support the feasibility of GaitSIT as a portable, low-cost, and immersive assessment framework for characterizing sensory-condition-specific gait–balance responses after accounting for walking speed and providing indirect behavioral indices related to sensory reweighting.

## 1. Introduction

Maintaining upright balance during locomotion depends on the central nervous system’s (CNS’s) ability to integrate multisensory inputs—somatosensory, visual, and vestibular—to generate appropriate motor outputs. The somatosensory system detects information from walking surfaces and joint position via the feet and limbs, the visual system provides spatial information from the environment, and the vestibular system senses head motion and orientation relative to gravity. These inputs converge in the CNS and are processed to construct an internal model of body orientation and motion—a process known as sensory integration [1]. Critically, during sensory integration, the CNS constantly adjusts the relative contributions of multisensory inputs depending on changing environmental demands and input reliability. This adaptive process—termed sensory reweighting—supports real-time corrections in gait–balance [2,3,4].

Several clinical posturography frameworks have been developed to systematically manipulate the availability and reliability of visual and somatosensory inputs in order to evaluate sensory integration and sensory reweighting during static balance control. Computerized dynamic posturography (CDP), including the Sensory Organization Test (SOT), remains a reference framework for probing sensory use in standing (quiet) balance. In the SOT, support-surface and visual-surround cues are manipulated across the canonical six conditions—the standard 2×3 matrix that crosses three visual contexts (eyes open, eyes closed, sway-referenced surround) with two surfaces (firm, sway-referenced support) [5]. In sway-referenced surround and sway-referenced support conditions, participants attempt to maintain a quiet stance while the visual surround or the support surface moves in proportion to their body sway, rendering visual input (surround) or somatosensory input (support surface) unreliable for postural control. Recent work refines the SOT evidence base with age-stratified norms and meta-analytic syntheses, emphasizing consistent calibration and purporting to improve the comparability of sway measures across clinical and research applications [6]. In addition, NeuroCom’s modified Clinical Test of Sensory Interaction on Balance (mCTSIB) is a clinically useful tool utilized for assessing the sensory contributions (somatosensory, visual, and vestibular) to an individual’s quiet balance. The mCTSIB assesses an individual’s static balance by completing four separate conditions: eyes open and eyes closed on a firm surface, followed by eyes open and eyes closed on a foam surface [7]. However, because most falls occur during movement rather than a quiet stance, there is a need for locomotion-focused assessments that apply SOT-style or mCTSIB principles while individuals are walking.

To extend SOT principles to gait, the Locomotor SOT (LSOT) introduced treadmill-based optic-flow and support-surface manipulations and demonstrated that sway variability during walking reflects SOT-like sensory organization patterns observed in standing balance, with greater visual influence during gait compared with stance [8]. However, the LSOT remains inaccessible in clinical settings due to its high cost, large size, immobility, and complexity. Specifically, the LSOT requires an instrumented treadmill, motion-capture, synchronized optic-flow projection environment, and laboratory-based support infrastructure, limiting its portability and routine clinical implementation. To address the limitation of the SOT as a static balance assessment and the limited clinical accessibility of the LSOT, we evaluated the Gait Sensory Interaction Test (GaitSIT)—a novel tool that integrates a low-cost head-mounted display (HMD)-based virtual reality (VR) system with foam surfaces to assess gait–balance by systematically manipulating somatosensory and visual inputs. The GaitSIT, therefore, preserves overground locomotion, allowing participants to walk through a physical environment within a clinically feasible and space-efficient framework. Commodity HMDs have been shown to enable controlled visual perturbations and head-level kinematic measurements at relatively low cost. Validation studies report strong agreement between consumer HMD tracking and laboratory motion capture, and close correspondence between HMD-derived head sway and force-plate center of pressure under visual manipulations [9,10]. Feasibility work further demonstrates that HMD-derived measures scale with visual disturbance intensity during stance and short walking tasks and relate to mobile-posturography measures, supporting HMD-based systems for portable balance screening [11]. These studies support the use of HMD technology within the GaitSIT as a valid, portable, and clinically feasible approach for assessing sensory-related gait–balance behavior during overground walking.

Virtual reality-based balance paradigms allow precise, repeatable manipulation of visual environments and task demands (e.g., moving scenes, optic flow, virtual perturbations), enabling multisensory postural control to be probed in ways that go beyond traditional mechanical posturography [12] and thereby directly supporting the rationale for GaitSIT. Validation studies have demonstrated that HMD systems are generally valid, feasible, safe, and relatively low-cost alternatives to force plates for detecting balance impairments and fall risk [13,14]. Prior locomotion studies show that altered visual information can influence gait stability, and that responses depend on perturbation characteristics, speed-control mode, and display modality [15,16,17]. Overground HMD walking studies further report increased spatiotemporal and trunk-related variability in VR relative to the real environment, and clinical populations demonstrate heightened visual dependence under optic flow [18,19,20]. The contributions of key studies related to balance assessment, sensory reweighting, and virtual reality-based gait paradigms are summarized in Table 1.

Despite these advantages, the accuracy and stability of HMD-derived kinematic measurements remain important methodological considerations when using consumer-grade VR systems for gait and balance assessment. A known limitation of HMD inside-out tracking is cumulative drift in absolute spatial position over distance and time, arising from the underlying visual–inertial state-estimation algorithm. Across a 6 m overground walk, this drift can manifest as low-frequency curvature of the reconstructed trajectory along the anterior–posterior axis, which may contaminate absolute-position metrics if uncorrected. Two mitigations are applied in the present protocol. First, the mediolateral (ML) signal is mean-centered per trial before sway computation, which removes any constant ML offset introduced by drift. Second, sensory ratios were computed within each session relative to the baseline (condition 1, C1), which may reduce the influence of common offsets across conditions but does not fully eliminate residual drift. The exact trial-specific drift magnitude over the 6 m walking distance was not directly quantified in this study; therefore, residual low-frequency drift may still influence absolute ML sway values and may appear as gradual curvature in the reconstructed walking trajectory. Validation work using the same headset family has reported sub-millimeter agreement with motion-capture reference systems for short tasks [9], supporting the use of HMD-derived head kinematics as the primary signal for the 6 m walking distance used in this present study.

Within this methodological framework, GaitSIT was developed as an overground extension of clinical sensory-interaction assessments for walking balance. Unlike the SOT and LSOT, GaitSIT preserves preferred-speed overground walking while applying the full 2 × 3 SOT sensory-condition framework during gait using commodity HMD tracking as the primary kinematic source. Specifically, the assessment combines firm and foam walking surfaces with HMD-controlled visual environments, including stable scenes, eyes-closed/dark conditions, and rotating visual scenes, to systematically manipulate somatosensory, visual, and vestibular contributions to walking balance. GaitSIT utilizes headset-derived position, velocity, and acceleration measures to characterize gait-related sway behavior and sensory-condition-specific changes in head-level walking balance under sensory-challenged walking conditions. The purpose of this present study was to evaluate the ability of GaitSIT to systematically perturb and quantify sensory-related changes in walking balance in healthy young adults. It was hypothesized that GaitSIT would demonstrate significant differences across sensory conditions and sensory ratios under standardized and ecologically relevant walking conditions.

## 2. Methodology

### 2.1. Participants

Healthy young adults (18–35 years) were recruited from the Clarkson University community to evaluate the ability of GaitSIT to systematically manipulate sensory conditions during walking, quantify the corresponding gait sway metrics and sensory ratios, and establish normative baseline data before including individuals at actual risk of falls. A priori sample size estimation indicated that 29 participants were adequate for a repeated-measures design involving 6 conditions across 3 GaitSIT sessions, assuming an effect size of 0.30, α=0.05, and statistical power of 0.80 (G*Power, Version 3.0.10) [23]. A medium effect size (0.30) was selected because GaitSIT is a novel assessment, and this conservative estimate is consistent with recommendations for exploratory repeated-measures designs and prior sensory-perturbation and VR-based balance studies demonstrating moderate condition-related effects on gait and sway outcomes [3,24,25]. Eligible participants were healthy young adults aged 18–35 years who were able to ambulate independently without assistive devices. Additional inclusion criteria required participants to complete six overground walking assessments on both firm and foam surfaces (each 6 m in length) during a two-day testing protocol lasting approximately 20–40 min. Participants were also required to have corrected visual acuity of at least 20/50 (Snellen) to ensure adequate perception of the VR stimuli presented through an HMD (i.e., Meta Quest 3). Participants’ biodemographic information, including age, height, weight, BMI, and sex distribution, is summarized in Table 2.

Individuals were excluded if they had a history of concussion, vestibular disorders, or balance/oculomotor impairments within the previous six months; neuropathic conditions affecting the lower extremities; or significant musculoskeletal impairments, including spinal or pelvic malalignment or leg length discrepancy, that could affect balance performance. Participants were also excluded if they had neck pain or restricted cervical range of motion, underwent orthopedic surgery within the previous six months that could influence postural control, had visual impairments preventing them from following VR cues, or had a pronounced susceptibility to motion sickness in immersive VR environments. This study was approved by the Clarkson University Institutional Review Board (protocol #25-08), and all eligible participants provided written informed consent prior to participation.

### 2.2. Measurement Tools

**Figure 1:**
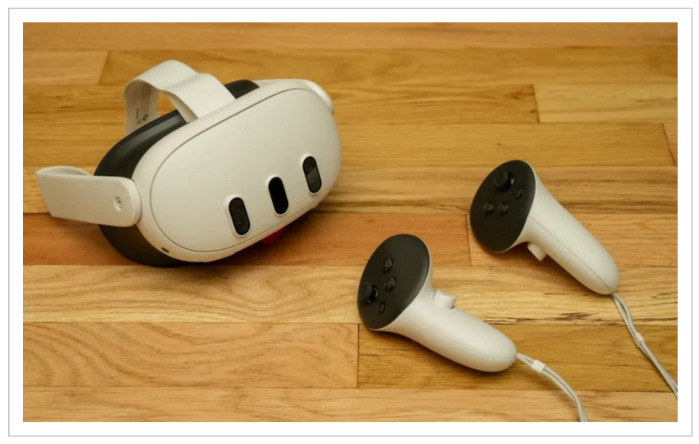
Meta Quest 3 VR headset used for GaitSIT application.

**Figure 2:**
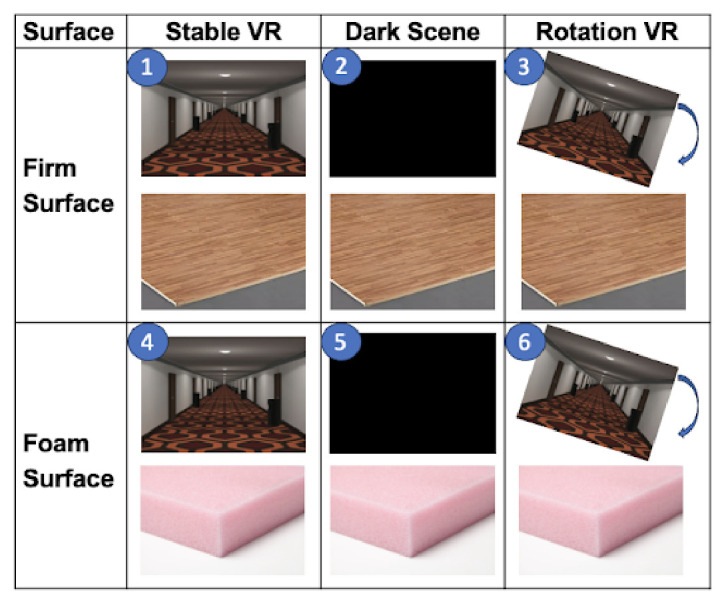
Gait Sensory Interaction Test (GaitSIT). Top pictures of firm and foam surfaces: VR hallway. Bottom pictures: brown floor (firm) and foam surfaces. Numbers represent conditions and arrows represent clockwise rotation. VR = virtual reality.

(a) Oculus Quest 3: The VR component of the GaitSIT uses the Oculus Quest 3 headset (Meta Platforms Inc., Menlo Park, CA, USA; Figure 1). The GaitSIT virtual environment is developed in Unity (C#) and presents a virtual hallway used to deliver standardized visual perturbations during overground firm and foam walking conditions. GaitSIT has six conditions designed to progressively challenge somatosensory, visual, and vestibular contributions to walking balance control. The six conditions (C) are: C1—firm surface, eyes open with a stable scene; C2—firm surface, eyes physically closed while the headset displayed a dark VR scene; C3—firm surface, eyes open with a rotating scene; C4—foam surface, eyes open with a stable scene; C5—foam surface, eyes physically closed while the headset displayed a dark VR scene; and C6—foam surface, eyes open with a rotating scene. In the rotating-scene conditions (C3 and C6), the virtual hallway rotated clockwise about the vertical (yaw) axis at 4°/s until reaching a maximum angular displacement of 45°, where it remained for the remainder of the trial. The headset’s built-in tracking system records head kinematics (position, velocity, and acceleration) during each test condition, and these data are used to calculate sway measures and sensory ratios. Figure 2 shows the six GaitSIT conditions (firm and foam surfaces combined with stable, dark, and rotating VR scenes).
(b) Gel Memory Foam Mats: The unstable walking surface was created using five gel memory foam toppers (Smiaoer, USA; 80 × 76 × 3 inches) placed end-to-end to form a continuous walkway. This surface was intended to mimic reduced somatosensory reliability similar to foam conditions used in clinical balance assessments such as the CTSIB.
(c) Modified Clinical Test of Sensory Interaction on Balance (mCTSIB): The mCTSIB was used as a clinical standing balance reference measure. It includes four conditions: (1) eyes open on a stable surface, (2) eyes closed on a stable surface, (3) eyes open on a foam surface, and (4) eyes closed on a foam surface. The test was administered using the NeuroCom^*R*^ SMART Balance Master 8.6.0; Clackamus, Oregon, USA extended force platform with a Neurocom (Equitest) foam pad (18 × 18 × 5 inches). Participants stood upright with feet shoulder-width apart and arms at their sides for 10 s per condition. Three trials were completed per condition. Outcome measures included equilibrium scores (sway velocity) and sensory ratios.
(d) Visual Analog Scale (VAS): A Visual Analog Scale (0 = no symptom, 10 = worst symptom) was administered before and after each test condition to rate headache, dizziness, nausea, and perceived instability. Participants reporting symptoms > 3 were withdrawn from further testing for safety reasons [26].

### 2.3. Procedures

The Oculus headset was securely positioned on the participants’ head to ensure a clear view of the VR environment. Participants performed the six GaitSIT conditions by walking straight barefoot at their preferred speed while looking straight ahead and maintaining a steady head position. For the dark-scene conditions, participants were instructed to physically close their eyes while the headset displayed a dark VR scene to eliminate residual visual cues from the headset display; compliance was monitored through standardized verbal instruction before each trial and assessor supervision throughout the walking task. Participants performed three sessions administered by two assessors (A1 and A2). These three sessions will allow for the evaluation of inter- and intra-reliability of the GaitSIT in a separate research publication. The first session was conducted by assessor A1 (A1.GaitSIT.1) and, after a 5 min rest, the second was conducted by assessor A2 (A2.GaitSIT.2). Approximately 24 h later, participants returned for the third GaitSIT session conducted by A1 (A1.GaitSIT.3). The GaitSIT application provided standardized audio and visual instructions at the start of each trial, minimizing assessor-related differences in trial instruction and administration. Furthermore, no familiarization trials were performed prior to testing, ensuring that all participants completed the assessment under the same standardized conditions. Within each session, the six GaitSIT conditions were completed in the same fixed sequence for all participants: C1, C2, C3, C4, C5, and C6. C1 was administered first because it served as the baseline reference for sensory-ratio calculations. Verbal ratings of headache, dizziness, nausea, and stability (0 = no symptom to 10 = worst symptom) were obtained immediately before and after each trial. Following a 5 min rest, participants completed the mCTSIB, administered by (A1.mCTSIB.1), to assess the concurrent validity of the GaitSIT. For the mCTSIB, participants stood barefoot with feet shoulder-width apart for 10 s in each of the four standard conditions. Each condition was performed three times, and the average was used for analysis. The mCTSIB was collected as a clinical standing-balance reference measure for future concurrent-validity analysis. These mCTSIB-based concurrent-validity findings, along with formal inter-/intra-rater reliability analyses of GaitSIT, will be reported separately in a forthcoming companion paper; the present manuscript focuses on the GaitSIT data-processing pipeline and condition-specific cohort outcomes.

### 2.4. Safety and Tolerability

The two assessors walked alongside the participant for every trial of the GaitSIT to provide physical guarding. A Visual Analog Scale (0–10) for headache, dizziness, nausea, and perceived instability was administered immediately before and after every condition solely to monitor participant safety and tolerability; these scores were not included in the statistical analyses. Any rating greater than 3/10 on any symptom resulted in immediate trial cessation, with subsequent retesting only after baseline ratings were re-established; if symptoms did not resolve within 10 min, the session was terminated. A 5 min seated rest was provided between sessions to mitigate vestibular fatigue and cumulative cybersickness, consistent with short-frequent-session dosing recommended for immersive VR [21]. To address the possibility of carryover sensory adaptation across conditions, C1 was always presented first (required as the baseline reference for sensory-ratio computation), and sessions were spaced by a 5 min rest within day and by approximately 24 h between day 1 and day 2 to allow recovery between assessment blocks.

### 2.5. Data Management

The primary data sources for this study include foundational time-series position, and velocity and acceleration data from the Oculus Quest 3 headset, measured in three orthogonal directions: medio-lateral (ML, X), vertical (Y), and anterior–posterior (AP, Z). The X, Y, and Z represent side-to-side, up–down, and forward–backward directions, respectively. The ML component was used as the primary indicator of lateral deviation from a straight walking path during each trial condition. The magnitude of ML deviation was used to quantify gait sway (i.e., walking balance control), and these sway measures were subsequently used to calculate sensory ratios.

#### 2.5.1. Data Preprocessing and Calculation of Mean Values

For data processing, HMD recordings were inspected for completeness and obvious artifacts. Trials with missing samples exceeding 100 ms, or tracking dropouts spanning more than 5% of the trial duration, were excluded; brief gaps (less than 100 ms) were filled by linear interpolation. Because the Oculus Quest 3 emits sensor-fused position, velocity, and acceleration through proprietary visual–inertial state-estimation algorithms, no additional post hoc low-pass filtering was applied; instead, we relied on the headset’s native tracking output, consistent with prior HMD-based balance assessments [27]. A second filtering stage applied to an already-smoothed signal would risk attenuation of condition-dependent peak and variability features. One preprocessing step was applied prior to sway computation: each trial’s ML (X) signal was mean-centered to remove any constant lateral offset introduced by start-position differences. After mean-centering, the resulting ML signal represented deviation around the trial-specific lateral midpoint and was used for all subsequent sway and sensory-ratio calculations. Across the 29-participant dataset used in this study, participant-wise plots of the processed ML time-series signals (by condition and session) were generated and visually inspected to verify that the traces were consistent and free of obvious tracking issues (e.g., abrupt jumps, long gaps, or unusual drift in the data). Visual inspection was used only as a final quality control step after applying the predefined criteria described above (exclusion of trials with missing samples exceeding 100 ms, exclusion of trials with tracking dropouts spanning more than 5% of the trial duration, interpolation of brief gaps less than 100 ms, and trial-level ML mean centering). Following preprocessing, summary kinematic values were computed for each trial along the ML direction for subsequent sway and sensory-ratio analyses.

#### 2.5.2. Sway Measure Calculation

One of the key indicators of dynamic balance control during gait is the ML sway, which reflects the deviations of the participant from a straight walking trajectory. To quantify this, we used mean position, velocity, and acceleration in the ML (X) direction as foundational kinematic variables. These deviations were further used to calculate continuous trajectory deviation angle and sensory ratios (i.e., somatosensory, visual, vestibular, and preference) for the GaitSIT using specific formulas. Continuous trajectory deviation angle is a sample-by-sample measure derived from movement trajectory coordinates that quantifies the instantaneous deviation of an individual’s path from the desired straight-ahead walking direction during locomotion. Somatosensory ratio measures the ability to use somatosensory input in the absence of vision, visual ratio measures reliance on visual input when somatosensory input is compromised, and vestibular ratio measures the ability to use vestibular input when both vision and somatosensation are unreliable or inaccurate. In addition, preference ratio measures the tendency to rely on visual input even when it is inaccurate, reflecting how well an individual can suppress misleading visual information and instead depend on vestibular and somatosensory cues for postural control.

Each sensory ratio is constructed so that the numerator represents a condition in which one or more sensory inputs are removed or made unreliable, while the denominator represents the reference condition (C1: firm surface, eyes open, stable scene), in which sensory cues are available. A ratio near 1 indicates head-level sway comparable to the baseline condition. A ratio greater than 1 indicates greater head-level sway under the manipulated condition relative to baseline, whereas a ratio less than 1 indicates lower head-level sway relative to baseline. The somatosensory ratio (C2/C1), visual ratio (C4/C1), vestibular ratio (C5/C1), and preference ratio ((C3 + C6)/(C2 + C5)) compare mean-centered ML head sway across their respective GaitSIT conditions. These ratios were used as indirect behavioral indices of sensory-condition-specific changes in head-level gait–balance responses, rather than direct measures of sensory-system contribution or neural sensory reweighting.

The sensory ratios are derived by using the following equations:

(1) $$\mathrm{Somatosensory}\;\mathrm{Ratio}=\frac{\frac{1}{N_{C2}}\sum_{n=1}^{N_{C2}}|x_{C2}\left[n\right]|}{\frac{1}{N_{C1}}\sum_{n=1}^{N_{C1}}|x_{C1}\left[n\right]|}$$

(2) $$\mathrm{Visual}\;\mathrm{Ratio}=\frac{\frac{1}{N_{C4}}\sum_{n=1}^{N_{C4}}|x_{C4}\left[n\right]|}{\frac{1}{N_{C1}}\sum_{n=1}^{N_{C1}}|x_{C1}\left[n\right]|}$$

(3) $$\mathrm{Vestibular}\;\mathrm{Ratio}=\frac{\frac{1}{N_{C5}}\sum_{n=1}^{N_{C5}}|x_{C5}\left[n\right]|}{\frac{1}{N_{C1}}\sum_{n=1}^{N_{C1}}|x_{C1}\left[n\right]|}$$

(4) $$\mathrm{Preference}\;\mathrm{Ratio}=\frac{\frac{1}{N_{C3}}\sum_{n=1}^{N_{C3}}|x_{C3}\left[n\right]|\;+\;\frac{1}{N_{C6}}\sum_{n=1}^{N_{C6}}|x_{C6}\left[n\right]|}{\frac{1}{N_{C2}}\sum_{n=1}^{N_{C2}}|x_{C2}\left[n\right]|\;+\;\frac{1}{N_{C5}}\sum_{n=1}^{N_{C5}}|x_{C5}\left[n\right]|}$$

For Equations (1)–(4), |x[n]| denotes the mean-centered ML signal for each condition; therefore, |x[n]| represents the absolute deviation from the trial-specific lateral midpoint rather than from the world origin. The mean absolute value was used to represent the average magnitude of lateral sway across the walking trial because it provides a straightforward measure of typical ML sway magnitude during overground walking and is less sensitive to isolated large deviations than RMS-based measures. Accordingly, the sensory ratios derived from these mean absolute ML sway values were interpreted as indirect behavioral indices of relative condition-related changes in head-level sway compared with the baseline condition.

These ratios were calculated for the GaitSIT (position, velocity, and acceleration), allowing us to compare the effects of somatosensory, visual, and vestibular manipulations on balance performance. To complement the primary measures of walking balance, we computed the continuous trajectory deviation angle, a sample-by-sample coordinate-based angular index derived from HMD position. This measure represents the angular relationship between lateral ML displacement and forward AP progression during the walking trial and was used as a descriptive index of trajectory deviation. By convention, positive angles indicate deviation toward +X (right) and negative toward −X (left), assuming forward progression along +Z.

(5) $$\theta_{\mathrm{Continuous}\;\mathrm{Trajectory}\;\mathrm{Deviation}\;\mathrm{Angle}}\left(t\right)=\tan^{-1}\;\left(\frac{x_{\mathrm{HMD}}\left(t\right)}{z_{\mathrm{HMD}}\left(t\right)}\right)\times \frac{180}{\pi }$$

Because this measure is based on the ratio of ML and AP coordinates, it may be influenced by the coordinate origin, anterior–posterior drift, and very small Z values near the start of the trial; therefore, it was interpreted descriptively as a trajectory-deviation index rather than as a direct measure of heading control. CTDA was calculated directly from the recorded mediolateral and anterior–posterior HMD coordinates at each sample. Because this measure is based on the ratio of ML and AP coordinates, it may be influenced by the coordinate origin, anterior–posterior drift, and very small Z values near the start of the trial; therefore, it was interpreted descriptively as a trajectory-deviation index rather than as a direct measure of heading control.

### 2.6. Statistical Analysis

To evaluate the effects of sensory manipulations on gait–balance, ML position, velocity, acceleration, and continuous trajectory deviation angle outcomes were analyzed across the six GaitSIT conditions (C1–C6) and three sessions (S1–S3) using linear mixed-effects models (LMMs). LMMs were selected because the present study uses repeated measurements (the same participants complete multiple conditions across multiple sessions), and the model accounts for within-participant dependence by including a participant-level random effect. For each outcome, fixed effects included condition, session, and their interaction (condition × session), with a random intercept specified for participants. Overall effects were evaluated using likelihood-ratio tests for condition, session, and condition × session. When significant effects were observed, post hoc pairwise comparisons were conducted (between conditions within each session and between sessions within each condition), and *p*-values were adjusted using Holm correction to control for multiple comparisons. Model-based estimated marginal means (EMMs) were computed for each session × condition cell to support interpretation and visualization. To examine the potential influence of walking speed on the primary GaitSIT outcomes, additional condition-specific analyses were performed separately for C1–C6. Within each condition, walking speed was entered as a covariate and session was included as a fixed factor. These analyses evaluated the association of walking speed with mediolateral position, velocity, acceleration, and continuous trajectory deviation angle.

Sensory ratios (somatosensory, visual, vestibular, and preference; SOM, VIS, VEST, and PREF) were analyzed using LMMs in two complementary ways. First, differences among sensory ratios within each session were examined using a linear mixed-effects model with RatioType (SOM, VIS, VEST, and PREF) specified as a fixed effect and participant included as a random intercept. When significant effects were detected, Holm-corrected post hoc pairwise comparisons (e.g., SOM vs VIS, SOM vs VEST, etc.) were performed to identify specific differences among ratios. Second, changes in each sensory ratio across sessions were evaluated by modeling each ratio as a function of session with a random intercept for participants. When significant session effects were observed, Holm-corrected post hoc comparisons were conducted among session pairs (S1 vs S2, S1 vs S3, and S2 vs S3). All data processing and statistical analyses were conducted using Python 3.13.5.

## 3. Results

In this cohort of 29 healthy young adults (mean age 24.9 ± 6.4 years), the GaitSIT systematically produced condition-dependent differences in headset-derived position, velocity, acceleration, and continuous trajectory deviation angle across the six GaitSIT conditions (C), with relatively consistent patterns across the three assessment sessions (S). After adjustment for trial-level walking speed, significant condition effects remained evident across all four primary GaitSIT outcomes, whereas session effects were not significant for any outcome. Generally, C1 and C2 demonstrated an upward trend from S1 to S2/S3, whereas C3 and C6 demonstrated a downward trend across the same sessions. Sensory ratios computed from positional sway showed decreasing visual and vestibular ratio values across sessions, alongside relatively stable somatosensory ratios.

### 3.1. Foundational Kinematic Data

Overall analyses demonstrated that the experimental conditions (C1–C6) differed significantly across the foundational kinematic measures of position, velocity, and acceleration (Table 3). There was a general trend of increasing kinematic values from C1 to C3, followed by a decrease at C4 and a subsequent increase from C4 through C6. Linear mixed-effects analyses revealed significant condition effects for position ($\chi^{2}\left(5\right)=86.207$, p<0.001), velocity ($\chi^{2}\left(5\right)=217.840$, p<0.001), and acceleration ($\chi^{2}\left(5\right)=91.288$, p<0.001). Relative to the baseline condition (C1), significant differences were observed across conditions C2–C6 for position, C3–C6 for velocity, and C2 and C5 for acceleration.

Session effects were significant for position ($\chi^{2}\left(2\right)=7.760$, p=0.021) and velocity ($\chi^{2}\left(2\right)=6.446$, p=0.040), but not for acceleration ($\chi^{2}\left(2\right)=1.043$, p=0.594). A significant condition × session interaction was observed for position ($\chi^{2}\left(10\right)=45.781$, p<0.001), whereas no significant interactions were identified for velocity ($\chi^{2}\left(10\right)=8.266$, p=0.603) or acceleration ($\chi^{2}\left(10\right)=7.748$, p=0.653). Taken together, condition significantly improved model fit for all three kinematic outcomes, whereas the contributions of session and the condition × session interaction differed across measures. Position showed significant session and interaction effects, velocity showed a significant session effect without a significant interaction, and acceleration showed no significant session or interaction effects.

**Figure 3:**
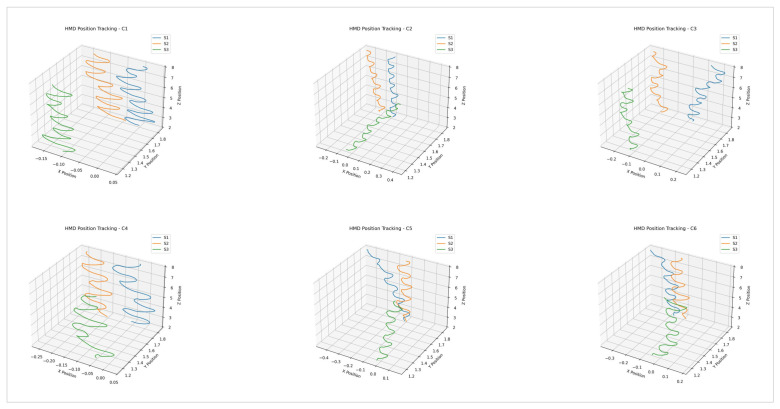
Position data for GaitSIT conditions. Representative data of position tracking data across six GaitSIT conditions for three assessment sessions. X = mediolateral, Y = vertical, Z = anterior–posterior, S = assessment session; the Z–direction has been shortened to clearly elicit the magnitude of deviation.

**Figure 4:**
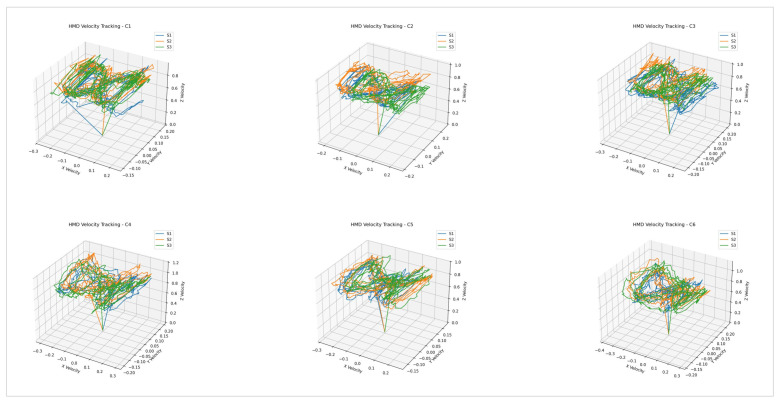
Velocity data for GaitSIT conditions. Representative data of velocity tracking data across six GaitSIT conditions for three assessment sessions. X = mediolateral, Y = vertical, Z = anterior–posterior, S = assessment session.

**Figure 5:**
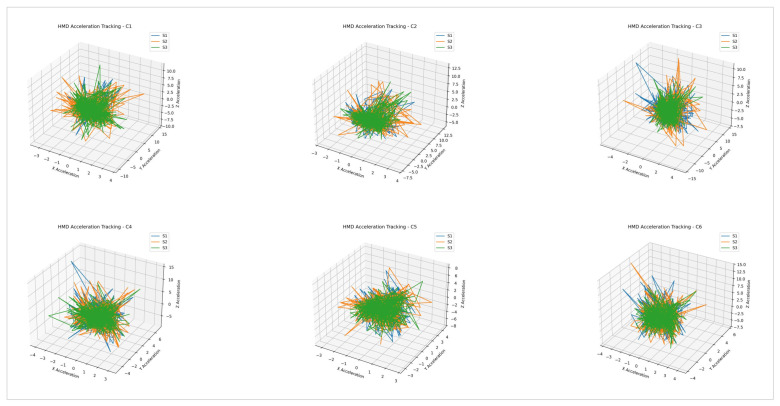
Acceleration data for GaitSIT conditions. Representative data of acceleration tracking across six GaitSIT conditions for three assessment sessions. X = mediolateral, Y = vertical, Z = anterior–posterior, S = assessment session.

To complement the group-level statistical analyses, representative trajectory visualizations from a healthy young adult participant (18-year-old male) were presented alongside cohort-averaged data to illustrate movement behavior across the six experimental conditions. Similar trajectory patterns were observed between the representative participant and the cohort averages for the three foundational kinematic measures across sessions. The collaged visualizations of HMD-derived trajectories demonstrated reproducible movement patterns across sessions for each condition (Figure 3, Figure 4 and Figure 5). Because the three-dimensional trajectory plots were intended primarily to illustrate movement behavior rather than provide quantitative comparisons, the main cohort-level patterns are summarized using the session-wise mean ± SE values in Table 3.

Visual inspection of the position trajectory plots showed condition- and session-specific trajectory patterns, including variations in mediolateral displacement and directional progression across conditions. Deviations from the nominal straight walking path and directional shifts were visible throughout the trajectory visualizations across conditions and sessions. Visual inspection also suggested similar overall trajectory patterns between the corresponding firm- and foam-surface condition pairs (C1–C4, C2–C5, and C3–C6).

Similarly, velocity trajectories demonstrated synchronized fluctuations across the three spatial axes throughout all sessions. Velocity values were lowest during C1, increased during C2 and C3, peaked during C4, decreased during C5, and increased again during C6. Comparable velocity progression patterns were observed between the representative participant and the cohort averages across sessions. Across repeated sessions, S1 exhibited relatively compact velocity traces, whereas S2 and S3 demonstrated broader trajectory distributions in selected conditions.

Acceleration trajectories also exhibited rhythmic movement patterns that remained similar across sessions for each condition. The representative participant and cohort-average acceleration trajectories demonstrated comparable spatial distributions across the X, Y, and Z directions. Session 1 demonstrated relatively scattered acceleration traces, whereas S2 and S3 exhibited comparatively more centralized trajectory distributions. Abrupt spikes and pronounced asymmetries were not consistently observed across conditions or sessions.

Overall, distinct movement behaviors were observed between test conditions, whereas trajectory patterns within each condition remained relatively consistent across sessions for both the representative participant and the cohort-average data. The observed trajectory patterns for position, velocity, and acceleration corresponded with the statistical findings across the six experimental conditions.

### 3.2. Continuous Trajectory Deviation Angle

**Figure 6:**
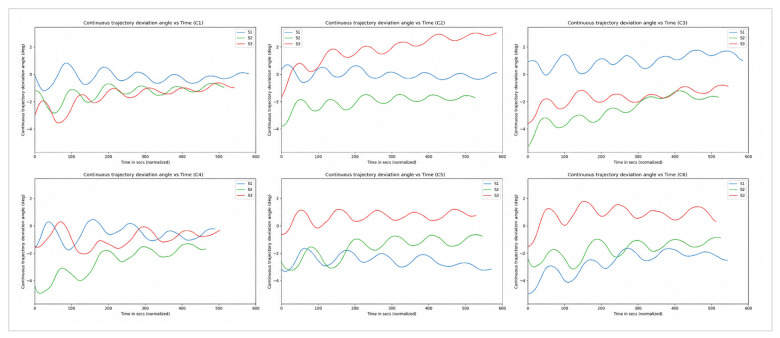
Continuous trajectory deviation angle across six GaitSIT conditions for three assessment sessions. S = assessment session.

Similar to the foundational kinematic measures, continous trajectory deviation angle (CTDA) differed significantly across the six experimental conditions (Table 3), generally increasing from C1 to C3, decreasing at C4, and increasing again from C4 through C6. Significant effects were observed for condition ($\chi^{2}\left(5\right)=70.258$, p<0.001), session ($\chi^{2}\left(2\right)=9.525$, p=0.009), and the condition × session interaction ($\chi^{2}\left(10\right)=40.922$, p<0.001). Pairwise comparisons revealed significant differences between the baseline condition (C1) and conditions C3–C6. These findings indicate that CTDA varied significantly across experimental conditions and sessions, with the effects of condition differing across testing sessions. Figure 6 presents representative session-wise CTDA traces across conditions C1–C6, while Table 3 summarizes the corresponding cohort-level mean ± SE values.

### 3.3. Influence of Walking Speed on Primary Outcomes

After adjusting for trial-level walking speed, the primary mixed-effects models showed significant main effects of condition for all four outcomes: position, F(5,480.75)=9.309, p<0.001; velocity, F(5,477.74)=40.726, p<0.001; acceleration, F(5,477.74)=8.935, p<0.001; and CTDA, F(5,477.78)=4.599, p<0.001. The main effects of session were not significant for any outcome, indicating that overall values did not differ significantly across the three testing sessions after accounting for walking speed. The condition × session interaction remained significant only for position, F(10,473.94)=4.436, p<0.001, but was not significant for velocity, acceleration, or CTDA. Walking speed was significantly associated with position, F(1,267.84)=43.049, p<0.001, acceleration, F(1,329.01)=31.670, p<0.001, and CTDA, F(1,297.29)=88.290, p<0.001, but not ML velocity, F(1,502.91)=3.676, and p=0.056. The corresponding fixed-effect estimates showed that walking speed was negatively associated with position (B=−0.234, SE=0.036, p<0.001), acceleration (B=−0.651, SE=0.116, p<0.001), and CTDA (B=−7.288, SE=0.776, p<0.001). The association between walking speed and velocity was not significant (B=−0.012, SE=0.006, p=0.056). Collectively, these results indicate that condition-related differences in GaitSIT outcomes persisted after adjusting for walking speed, although walking speed contributed meaningfully to position, acceleration, and CTDA.

### 3.4. Sensory Ratios

**Figure 7:**
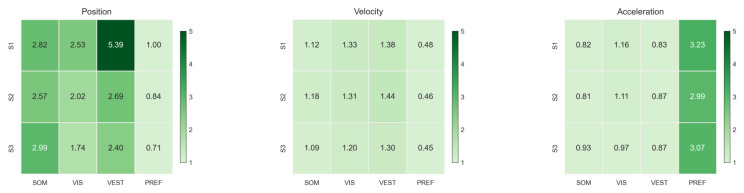
Session-wise average sensory ratio heatmaps across all participants for position, velocity, and acceleration metrics.

Sensory-ratio heatmaps (Figure 7) were examined to characterize session-wise sensory-ratio patterns across the cohort for the position, velocity, and acceleration metrics. Overall, sensory-ratio analyses showed metric- and session-specific differences among SOM, VIS, VEST, and PREF ratios across position, velocity, and acceleration outcomes.

For the position metric, visual (VIS) and vestibular (VEST) ratios generally demonstrated larger values relative to the somatosensory (SOM) and preference (PREF) ratios across sessions. Correspondingly, post hoc analyses identified significant differences between most sensory ratios. The primary exceptions included the SOM–VIS comparison in S1 (*p* = 0.581), as well as the SOM–VEST, SOM–VIS, and VIS–VEST comparisons in S2 (*p* = 0.978, 0.760, and 0.760, respectively) and S3 (*p* = 0.635, 0.353, and 0.635, respectively). Similarly, for the velocity metric, sensory ratios remained relatively comparable across sessions, with visual and vestibular ratios demonstrating similar magnitudes throughout all three sessions. Although significant differences were observed between most sensory ratios, nonsignificant comparisons were identified for the VIS–VEST comparison in S1 (*p* = 0.371) and S2 (*p* = 0.087), as well as the SOM–VIS and VIS–VEST comparisons in S3 (*p* = 0.164 and 0.217, respectively).

In contrast, acceleration sensory ratios remained relatively stable across sessions. Nevertheless, significant differences were still observed between most sensory ratios. Nonsignificant comparisons were limited to SOM–VEST across S1, S2, and S3 (*p* = 0.785, 0.215, and 0.661, respectively), together with SOM–VIS in S3 (*p* = 0.353) and VIS–VEST in S3 (*p* = 0.220).

## 4. Discussion

This study demonstrates a novel approach to the evaluation of postural control using the GaitSIT VR assessment, with a focus on analyzing the role of sensory inputs—specifically, somatosensory, visual, and vestibular input—in maintaining walking balance. Overall, GaitSIT produced significant condition-dependent changes across position, velocity, acceleration, and continuous trajectory deviation angle measures, while movement patterns remained relatively consistent across repeated sessions. Sensory-ratio analyses further demonstrated distinct responses across sensory conditions, with the largest effects observed for vestibular and visual ratios in the position metric. Collectively, these findings demonstrate the ability of GaitSIT to characterize sensory-related changes in gait and balance behavior within an immersive virtual reality environment.

### 4.1. GaitSIT Characterizes Condition-Dependent Changes in Position and Velocity Trajectories During Walking Balance

The observed condition effects across the six GaitSIT conditions within the foundational kinematic measures support the underlying sensory-interaction framework of the assessment [5]. Across the foundational kinematic outcomes, significant condition effects were observed for position, velocity, and acceleration, with progressively greater and more consistent deviations emerging from C1 through C6 as sensory conditions became increasingly challenging. GaitSIT is designed to extend the logic of the Sensory Organization Test (SOT) to walking by systematically manipulating somatosensory, visual, and vestibular inputs during locomotion through combinations of firm and foam walking surfaces, visual occlusion, and rotating visual scenes. Consistent with the SOT and Locomotor Sensory Organization Test (LSOT), the present findings demonstrated that altering the reliability of sensory inputs produced measurable changes in walking-related sway behavior [8]. The progressive changes observed across conditions further demonstrate the increasing sensory demands imposed from firm to foam walking conditions. As visual and somatosensory information became less reliable across conditions, greater mediolateral (ML) deviations were observed within the kinematic measures. Conditions involving foam surfaces and rotating visual scenes appeared to impose the greatest sensory demands because they simultaneously altered somatosensory feedback and visual orientation cues, thereby producing larger deviations relative to the baseline condition (C1), where all sensory inputs remained reliable. Among the foundational kinematic measures, position data demonstrated the most consistent condition-dependent changes, with significant differences observed across conditions C2–C6 relative to baseline. In contrast, acceleration demonstrated fewer significant condition-related differences. These findings suggest that the positional measure, rather than the velocity or acceleration measures, may be particularly sensitive to sensory-related changes in walking balance during the GaitSIT paradigm.

Although significant session effects were identified for position and velocity, movement patterns remained relatively comparable across repeated sessions. In addition, only the position metric demonstrated a significant condition × session interaction, whereas velocity and acceleration did not. These findings suggest that the observed variability across sessions was not primarily driven by substantial between-session inconsistency. The GaitSIT protocol incorporated standardized audio and visual instructions delivered through the application, and no familiarization trials were performed prior to testing, thereby promoting procedural consistency across sessions. However, because the three GaitSIT sessions differed by assessor and testing day, the observed session effects may reflect a combination of familiarization, assessor-related differences, same-day retesting effects, and day-to-day variability rather than adaptation alone. As these factors were not statistically separated in the present analysis, the source of the session effects cannot be determined conclusively. Nevertheless, the relatively stable waveform morphology and trajectory progression patterns observed across sessions, together with the limited number of significant session and interaction effects, support the overall reproducibility of the GaitSIT paradigm. Formal inter- and intra-rater reliability analyses will be reported separately.

### 4.2. Continuous Trajectory Deviation Supports the GaitSIT’s Ability to Characterize Sensory-Specific Directional Control During Walking

The continuous trajectory deviation angle findings support the ability of the GaitSIT to systematically perturb and objectively quantify directional control behavior during sensory-challenged walking. Significant condition effects, particularly between C1 and C3–C6, demonstrated that the sensory manipulations incorporated within the GaitSIT elicited distinct changes in walking trajectory regulation and directional stability. These findings are consistent with the study hypothesis that gait–balance metrics would differ across sensory conditions and further support the GaitSIT framework as a walking-based sensory interaction assessment. The observed oscillatory waveform behaviors likely reflect continuous corrective adjustments used to maintain forward progression and stability under altered sensory conditions, consistent with sensory reweighting principles during locomotion [2,28].

Despite significant session and condition × session effects, waveform morphology remained relatively stable within conditions across sessions, suggesting that the observed variability was not primarily attributable to poor between-session reproducibility. Rather, the session-related differences may indicate progressive refinement of directional control strategies with repeated exposure to the sensory-challenging walking conditions. The comparatively shorter waveform traces observed in later sessions may reflect adaptations in directional control behavior and reduced responsiveness to the sensory perturbations following repeated exposure to the virtual environment [29]. These findings therefore support the reproducibility of the GaitSIT paradigm while also suggesting sensitivity to adaptive locomotor responses during repeated sensory perturbation.

Compared with the foundational position and velocity findings, the continuous trajectory deviation angle provided complementary information regarding the continuous temporal characteristics of directional regulation during walking. Whereas position and velocity metrics characterized overall gait–balance behavior, the continuous trajectory deviation angle captured dynamic directional adaptations throughout locomotion. Together, these findings support GaitSIT as a portable and ecologically relevant tool for characterizing complementary dimensions of sensory-condition-specific walking-balance behavior.

### 4.3. Walking Speed Influences GaitSIT’s Position, Acceleration, and CTDA Outcomes

The speed-adjusted findings strengthen the interpretation that the GaitSIT conditions elicited condition-specific gait–balance responses that were not explained solely by differences in walking speed. This is important because walking speed is known to influence gait biomechanics broadly, including spatiotemporal parameters, kinematics, kinetics, and ground reaction forces [30], and recent work further shows that mediolateral stability measures can vary systematically with gait speed and individual preferred-speed characteristics [31]. The persistence of significant condition effects across ML position, velocity, acceleration, and CTDA therefore supports the sensitivity of the VR sensory perturbation paradigm to sensory-condition demands. At the same time, the significant association of walking speed with position, acceleration, and CTDA indicates that these outcomes should be interpreted as speed-sensitive gait–balance measures rather than purely sensory-control outcomes. The negative walking-speed coefficients further indicate that faster walking speed was associated with lower position, acceleration, and CTDA values after accounting for condition, session, and their interaction. This suggests that slower walking may be accompanied by greater lateral deviation, higher acceleration-related instability, and larger trajectory deviation, reflecting a more cautious or less dynamically stable gait pattern under sensory-challenging conditions. This interpretation is consistent with recent VR gait studies showing that visual/optic-flow manipulations can alter walking behavior, including gait speed and stability-related adaptations, particularly when visual feedback is perturbed through immersive or semi-immersive VR systems [17,32]. Collectively, these findings suggest that GaitSIT captures meaningful sensory-condition-related changes in walking balance while also highlighting the need to account for walking speed when interpreting head-derived gait stability outcomes in VR-based balance assessment.

### 4.4. Sensory Ratios Provide Indirect Behavioral Indicators Related to Gait-Specific Sensory Reweighting During Walking

The sensory-ratio findings support the primary objective of this study by demonstrating that GaitSIT can systematically perturb visual and somatosensory inputs and characterize associated changes in head-level gait–balance responses in healthy young adults. Consistent with the study hypothesis, significant differences were observed across most sensory ratios and outcome measures, indicating that sensory manipulations successfully elicited measurable changes in gait–balance behavior. These findings provide additional support for the sensory-reweighting framework underlying the GaitSIT paradigm, whereby the central nervous system dynamically adjusts the relative contributions of somatosensory, visual, and vestibular inputs according to changing environmental demands and sensory input reliability during locomotion [2,3,4]. Together, these findings support the GaitSIT framework as a walking-based sensory interaction assessment that characterizes sensory-condition-dependent changes in head-level gait–balance responses and provides indirect behavioral indicators related to sensory reweighting during locomotion [33].

For the position metric, the generally larger visual and vestibular ratios relative to the somatosensory and preference ratios indicated greater relative changes in head-level ML sway under the corresponding GaitSIT conditions. This is consistent with the previous literature demonstrating the important role of visual–vestibular integration in dynamic gait stability during locomotion [28,34]. Similarly, the relatively comparable visual and vestibular ratio magnitudes observed for the velocity metric indicated similar relative patterns across these two condition-based indices. In contrast, acceleration sensory ratios remained comparatively stable across sessions despite significant between-ratio differences. This may suggest that acceleration-based responses were less sensitive to repeated sensory exposure than positional and velocity sway measures. Together, these findings indicate that different kinematic domains capture distinct aspects of sensory-condition-specific gait–balance responses during walking. However, because the sensory ratios were derived from HMD-based head sway, they should be interpreted as indirect behavioral indices of sensory-condition-specific head-level gait–balance responses, rather than direct measures of sensory-system contribution or neural sensory reweighting; these indices may also be influenced by walking speed, head-control strategy, and residual trajectory drift.

The relatively consistent sensory-ratio patterns observed across sessions further support the reproducibility of the GaitSIT paradigm. Although some nonsignificant comparisons were observed between visual and vestibular ratios, these similarities may reflect the interdependent roles of visual and vestibular processing during dynamic balance control rather than poor condition differentiation. Overall, these findings support GaitSIT as a portable and ecologically relevant tool for characterizing sensory-condition-specific changes in head-level walking balance.

### 4.5. Limitations and Future Directions

The findings of the present study should be interpreted within several limitations. First, the study included only healthy young adults. Future studies should evaluate GaitSIT in older adults and in individuals with vestibular, neurological, or musculoskeletal impairments to determine its clinical sensitivity, feasibility, and generalizability in populations at increased risk of walking-balance deficits. Nevertheless, the ability of GaitSIT to demonstrate consistent condition-dependent differences and reproducible indirect sensory ratio patterns provides important foundational evidence supporting its potential utility for characterizing sensory-condition-specific changes in walking balance in clinical populations. Second, although GaitSIT demonstrated condition-dependent differences across the sensory manipulation, formal inter- and intra-rater reliability analyses and concurrent validity comparisons with the mCTSIB will be reported separately in a companion paper. Third, because the conditions were administered in a fixed order, potential adaptation or carryover effects from repeated exposure to the VR and sensory perturbations could not be isolated from condition-related effects in the present study. Next, although the Oculus inside-out tracking system provides built-in spatial stabilization, small positional inaccuracies inherent to consumer-grade HMD tracking may still have influenced the kinematic measurements.

In addition, HMD-derived head kinematics provide a practical and low-cost proxy for postural sway during gait, but do not directly quantify whole-body center-of-mass dynamics or lower-limb mechanics such as step width, step length, double-support time, and margin of stability. Accordingly, GaitSIT outcomes should be interpreted primarily as measures of head-level sensory-conflict responses during walking rather than complete representations of whole-body postural control. Accordingly, the analyses were conducted using the native mean-centered HMD-derived signals, and comparisons with alternative preprocessing pipelines (e.g., detrending or conservative low-pass filtering) were not performed. Future studies may examine the influence of different preprocessing strategies on these outcomes.

Future studies should evaluate the sensitivity of the GaitSIT in populations with impaired sensory integration, including individuals with vestibular disorders, stroke, Parkinson disease, mild traumatic brain injury, peripheral neuropathy, and older adults at increased fall risk. Additional work will examine the responsiveness of GaitSIT outcomes to rehabilitation interventions and explore whether sensory-ratio profiles can help guide individualized balance-training strategies.

## 5. Conclusions

This study introduces a VR-based walking sensory interaction assessment, the GaitSIT system, that systematically manipulates visual and somatosensory conditions during overground walking to characterize sensory-condition-specific changes in walking balance under ecologically relevant conditions. The findings demonstrated significant condition-dependent differences across headset-derived position, velocity, acceleration, continuous trajectory deviation angle, and sensory-ratio measures, supporting the study hypothesis that the GaitSIT would detect measurable changes across sensory-challenging walking conditions. Importantly, condition-related differences in the primary kinematic outcomes persisted after adjusting for trial-level walking speed, although walking speed contributed to position, acceleration, and CTDA outcomes and should be considered when interpreting these measures. In addition, movement patterns, waveform morphologies, sensory-ratio profiles, and kinematic trends remained relatively consistent across repeated sessions, supporting the reproducibility of the paradigm while also suggesting adaptive locomotor responses to repeated sensory exposure. Collectively, these findings support the potential of GaitSIT as a low-cost, portable, immersive, and clinically feasible overground assessment framework for characterizing sensory-condition-specific changes in head-level walking balance. The sensory-ratio findings should be interpreted as preliminary and indirect behavioral indices of sensory-condition-specific head-level gait–balance responses that may be related to sensory reweighting, rather than direct measures of sensory-system contribution or neural sensory reweighting.

## Figures and Tables

**Table 1:** Summary of related work.

Paper	Brief Description
Ashida & Fujimoto [10]	Compares HMD head motion with force-plate CoP during vection and finds responses are statistically indistinguishable aside from gain, validating head motion as a practical CoP substitute.
Chien et al. [8]	Introduces the Locomotor SOT (LSOT) by extending SOT conditions to walking and demonstrates similar sensory pattern contributions but stronger visual influence during gait, positioning LSOT to probe gait sensory reweighting.
Craig et al. [9]	Validates Oculus/Quest head tracking for balance assessment: head tracking matches motion capture (ICC >0.99, mm-level error), and aligns with force-plate CoP, supporting low-cost portable posturography.
Horsak et al. [19]	Compares overground room-scale VR walking with real-world walking using 3D gait analysis. VR increases mediolateral trunk-velocity variability and strengthens COM–step-width coupling, while a slight reduction in anteroposterior margin of stability reflects adaptive control to reduced optic-flow reliability.
Horsak et al. [18]	Examines full-body biomechanics in immersive VR rooms of different sizes and finds modest speed reduction but marked increases in kinematic and step-width variability, independent of room size—indicating a conservative gait strategy.
Liu et al. [21]	Compares immersive versus non-immersive VR for peripheral vestibular dysfunction and finds immersive VR yields greater vertigo reduction; short, frequent sessions produce the best outcomes, informing VR-based rehab dosing.
Martelli et al. [22]	Examines gait responses to a virtual environment (VE) and to superimposed visual oscillations during overground walking. The VE shortens stride and increases stride-width variability; medio-lateral perturbations are most destabilizing, with partial adaptation over minutes, suggesting potential applications for gait assessment rather than treatment.
Perucca et al. [6]	Provides SOT norms for adults aged 80–89 and documents the greatest decline in vestibular-mediated control with advanced age, supplying reference values for the “very old.”
Rosiak et al. [11]	Investigates an immersive “ship-at-sea” virtual reality paradigm using the Meta Quest 2 HMD across firm and foam stance as well as a short 3 m walk. Head-sway velocity, obtained from the HMD’s integrated motion tracking, scales with the level of visual disturbance and correlates with measurements from a lumbar-mounted mobile-posturography sensor, supporting a portable and low-cost approach to balance screening.
Shelton et al. [16]	Tests ML optic flow at fixed versus self-paced treadmill speeds; fixed speed widens steps and increases local instability, while self-paced walking slows slightly to preserve stability—supporting self-paced protocols for diagnostics.
Song et al. [20]	Evaluates visual-perturbation sensitivity in chronic ankle instability (CAI) and shows larger increases in gait variability than controls under continuous ML optic flow, indicating greater visual dependence and limited reweighting.
Wilson et al. [17]	Compares HMD with an immersive room under pseudo-random AP/ML perturbations during self-paced walking; ML perturbations reduce stability in both, with larger effects under the HMD and increased speed-control variability.
Keshner et al. [12]	Reviews the evolution from computerized dynamic posturography (CDP) to virtual reality (VR) approaches for balance assessment, arguing that VR enables richer, more functional sensory conflicts. It highlights how optic-flow manipulations can induce visual–vestibular mismatch and vection to challenge postural control, and emphasizes pairing VR with objective measures (e.g., force plates or motion analysis) to quantify balance responses.
Soltani et al. [13]	Reviews 19 studies of head-mounted display (HMD) VR in older adults and finds it feasible for balance assessment/training, often distinguishing healthy from balance-impaired individuals and prompting more cautious task performance. It also notes generally low study quality and emphasizes the need for age-specific protocols and careful safety/usability considerations before broader clinical use.
Sylcott et al. [14]	Compares head-sway metrics from a consumer VR headset (HTC Vive) with force-plate center-of-pressure during quiet standing (eyes open/closed). Reports meaningful correlations and comparable test–retest reliability for common sway measures (e.g., RMS, peak-to-peak), supporting headset-based posturography as a low-cost alternative to force plates.

**Table 2:** Participant characteristics (*n* = 29).

Characteristic	Value
Age (years)	24.9 ± 6.4
Height (m)	1.72 ± 0.12
Weight (kg)	76.7 ± 16.1
BMI (kg/m^2^)	25.8 ± 3.8
Sex (male/female), n (%)	17 (58.6%)/12 (41.4%)

Values are mean ± SD unless otherwise noted.

**Table 3:** Session-wise average mean ± standard error values for position, velocity, acceleration, and continuous trajectory deviation angle (CTDA) across six conditions.

Session	Condition	Position (Mean ± SE)	Velocity (Mean ± SE)	Acceleration (Mean ± SE)	CTDA (Mean ± SE)
S1	C1	0.0656 ± 0.0014	0.0881 ± 0.0033	0.8732 ± 0.1511	1.7561 ± 0.0314
S1	C2	0.1425 ± 0.0035	0.0969 ± 0.0031	0.6840 ± 0.0483	1.6260 ± 0.0276
S1	C3	0.3244 ± 0.0047	0.1174 ± 0.0035	0.7893 ± 0.0266	3.8854 ± 0.0344
S1	C4	0.1186 ± 0.0026	0.1182 ± 0.0045	1.0291 ± 0.1647	2.0438 ± 0.0453
S1	C5	0.2538 ± 0.0051	0.1217 ± 0.0030	0.6454 ± 0.0187	2.8400 ± 0.0374
S1	C6	0.2832 ± 0.0048	0.1379 ± 0.0041	0.9437 ± 0.0644	3.8686 ± 0.0614
S2	C1	0.1056 ± 0.0014	0.0869 ± 0.0028	0.7931 ± 0.0793	1.3651 ± 0.0243
S2	C2	0.2085 ± 0.0049	0.0998 ± 0.0029	0.6199 ± 0.0204	2.2807 ± 0.0340
S2	C3	0.1881 ± 0.0043	0.1110 ± 0.0035	0.7848 ± 0.0294	2.1733 ± 0.0394
S2	C4	0.1659 ± 0.0023	0.1135 ± 0.0040	0.8508 ± 0.0682	2.1420 ± 0.0378
S2	C5	0.2300 ± 0.0042	0.1221 ± 0.0031	0.6642 ± 0.0209	2.6339 ± 0.0354
S2	C6	0.2112 ± 0.0045	0.1288 ± 0.0042	0.9212 ± 0.0797	4.2161 ± 0.0560
S3	C1	0.1223 ± 0.0018	0.0908 ± 0.0030	0.8616 ± 0.1289	1.5383 ± 0.0277
S3	C2	0.1969 ± 0.0042	0.0986 ± 0.0039	0.7613 ± 0.0855	2.1786 ± 0.0303
S3	C3	0.1620 ± 0.0039	0.1082 ± 0.0034	0.7816 ± 0.0303	1.9156 ± 0.0367
S3	C4	0.1279 ± 0.0023	0.1084 ± 0.0033	0.7615 ± 0.0281	1.6366 ± 0.0349
S3	C5	0.1776 ± 0.0035	0.1153 ± 0.0031	0.6768 ± 0.0216	1.9174 ± 0.0301
S3	C6	0.1775 ± 0.0034	0.1281 ± 0.0036	0.8405 ± 0.0312	2.0959 ± 0.0339

S = Session. C = Condition.

## Data Availability

The datasets generated and analyzed during the current study are not publicly available at present due to ongoing related research and publication efforts. The data will be released publicly after the completion of these associated publications.
